# Hydroleine—Hydrated Oil

**Published:** 1880-09-20

**Authors:** J. H. Egan

**Affiliations:** Tenn.


### Southern Medical Record.

EDITORS:

T. S. POWELL, M.D. W. T. GOLDSMITH, M.D. R. C. WORD, M.D.

*R. C. WORD, M.D., Managing Editor.*

☞All Communications and Letters on Business connected with the RECORD must be addressed to the Managing Editor.

VOL. X. ATLANTA, GA., SEPTEMBER 20, 1880. No. 9.

### ORIGINAL AND SELECTED ARTICLES.

### HYDROLEINE—HYDRATED OIL.

BY J. H. EGAN, M.D., TENN.

The credit of the introduction of cod-liver oil and its various combinations in the treatment of phthisis and all other diseases where emaciation is a prominent symptom, is due to the late Dr. John Hughes Bennett, of Edinburgh. It was soon discovered that while the exhibition of one or two bottles was advantageous and served to increase weight, yet its continued administration had the contrary effect. The stomach became disordered, eructations were produced, and the remedy had to be discontinued.

This arises from an overworked liver. The largest and most fatty liver is found in persons who shew the greatest emaciation.

The lungs are placed between the veins and arteries. No venous blood is allowed access to the arterial system until it has passed through the lungs and been oxygenated. The liver is placed between the food supply and the pulmonary circulation, intercepting every particle of food that can be absorbed by the veins. Everything that can be absorbed by the lacteals and lymphatics is conveyed by the thoracic duct to the lungs, and never reaches the liver. All the venous blood, except that charged with new food, is kept out of the reach of the liver.

and conveyed to the lungs. In fine, all fats not absorbed by the portal system of veins, all the products of interstitial nutrition are carried direct to the pulmonary circulation, but all other elements must be submitted to the operation of the liver before being fit for use.

The bile is manufactured by the liver from the fat, and only the excess can reach the hepatic vein. An accumulation of oil in the hepatic vein indicates that the liver has been overtaxed. The continued overtaxing of the liver renders it fatty, and in proportion as this occurs so the liver becomes more and more unable to perform its functions. We see then why for a time an emaciated patient improves under the use of oil, and why so soon as the liver becomes incapacitated from performing its function emaciation increases.

For a time it is possible to overtax any organ, and should the strain be removed, the organ at once recovers its normal state. As a corollary so soon as we find that the ingestion of oil disagrees with the patient, we ought at once to stop its use and stimulate the liver.

The best hepatic stimulants I have found are :

| | |
|---|---|
| Elixir wahoo and wahoo and blue flag or podophyllin.... | gr. 1 |
| Alcohol........................................ | ℨ ½ |
| Fluid extract aromatic powder.......................... | ℨ ½ |

A teaspoonful of either to be taken at night.

Fat is the substance we desire to exhibit, and the point with the physician is to administer in such a way that it will be taken up by the lacteals and lymphatics without straining the powers of the liver. If fat be not ingested then, as the demand for fat must be met, the system lives upon itself, producing the emaciation we desire to check.

In consumption, long before there has been a deposit of the tubercle, the fact that strikes the patient is his increasing weakness and emaciation. We make a critical examination and we are unable to explain it, as we find no disorder in any organ and, therefore, we are sure that, unless instant treatment be adopted, phthisis will result. It is in this stage that the disease is always curable.

Dissatisfaction with the action of cod-liver oil led the London physician to enquire into the matter, and the result of many years of research and experiment has resulted in placing before the profession "hydroleine." In Great Britain this preparation has supplanted both cod-liver oil and its combinations.

It is partially digested oil with water, and is sometimes denominated "hydrated oil." It is pleasant in its taste and never disagrees with the stomach. It can be exhibited indefinitely, and the immediate effect is increased weight and appetite, enabling the patient to take exercise and by that means burn up the waste tissue.

This is the great benefit derived from outdoor exercise; the chest becomes expanded, and fuller inspirations are made. Of my own personal experience I can state that I have never seen "hydroleine" or "hydrated oil" exhibited without the patient at once being cognizant of its beneficial effects.

